# Supplementary material for: Detection of Fused Genes in Eukaryotic Genomes using Gene deFuser: Analysis of the Tetrahymena thermophila genome
Source: BMC Bioinformatics. 2011 Jul 11;12:279. doi: 10.1186/1471-2105-12-279 (PMC3143110; doi:10.1186/1471-2105-12-279)
Supplement: Additional file 1 — Results of Gene deFuser for the Tetrahymena thermophila genome. This zip file contains the raw results of the analysis of the Tetrahymena genome using Gene deFuser. To view the contents, unzip the file and open the Final_Tet.html file in the resulting folder. [file 1471-2105-12-279-S1.ZIP › Results/3794.m00173.html]

Gene deFuser -- Results of Job Final\_Tet

 


Gene deFuser

| Home | Retrieve Results | References | Help |
| --- | --- | --- | --- |

Back to Main Results of Job Final\_Tet

# Query Name: 3794.m00173

Candidate fusion gene

## Query Sequence:

MQVRNQLKIQNSKKRDTAQQQIAKSERHNLSEQQIQIQNNQMTELSTQLMKIQGGGDTPAPRFGHTLTLISKSKAVLFGGAIGDSGKFIITNETYIFDYELKKWKKLECTGDIPSQRAAHASCQIDNMTMVIYGGAASGGGGLSNDELYLLDLKQYDSNDKSTQNGHYIKVPTSGPTPGKRYGHTMVYSKPHLIVFGGNTGTIPVNDVWVLNLEKGPYQWQKQCINAAEVPAVRVYHSASLCQTGSANGMMVVFGGRTQDQSPLNDTWGLRRHRNGMWDWVLAPYKQNSAILPVCRYQHRIEFIGPLMLVIGGRTSGGEEQGKCVEVYDTESSDWYRVNSFNRYRHSTVVIDSTMYIHGGFEPEFPNKPLDSMITIDLNQISYLFPKLGKHLGVKDFNNILGERNQIDKPASSPSNQLNQQQLQAQQQNSNMFNQQTFSKKQMQNQANLLQPQNQNTNNTKAYQQTLSNQMLDISIGGIQKVQLKQLNNAQRSYTPIKQQKNNKEIRLSNQVVIARTQDSENMNQYYNMNNYQQQQNFYGGIKKVNILDLQEESKKLIPQNQNDHSMMLVSPVDQNTTSLAEMFINSLMKKPEEWKHTELRLPFKKEFILRLCDEVQQIVLDQPSLLRLRIPLKIFGNIHGRFGDLLRFFGNFGFPVDEEGGDIEKFDYLFLGNYVDRGFNSLETVILLFALKLKYPDQIHLIRGAHEERRVNRLFGFGEECAVRLNEDINDPTSAYQRINRVFDSLPLGALIEEKIFCVHGGLGPHVTHVNEIVDIKRPVSPFSNPVVYDLLYSDYVENDPLPVSENKAKDYFSTGQTFKYSEAKVERFLEETGLNMIIRSKECVFDGFSRSQSGNVTTITSCSDYMGKYGNSACCIRIKKNFAIEPNIISRPQFRNGNWVGAENPVKTNLYFTEEEQKIRRRLASPLQKQY

### Significant Ortholog Group Hits and their Scores:

| N terminus | | C terminus | |
| --- | --- | --- | --- |
| [R] KOG4693 Uncharacterized conserved protein, contains kelch repeat | 8.21176291987806 | [DT] KOG0373 Serine/threonine specific protein phosphatase involved in cell cycle control, PP2A-related | 40 |
| [R] KOG0379 Kelch repeat-containing proteins | 6.76583794627394 | [R] KOG0375 Serine-threonine phosphatase 2B, catalytic subunit | 40 |
|  |  | [GT] KOG0372 Serine/threonine specific protein phosphatase involved in glycogen accumulation, PP2A-related | 40 |
|  |  | [T] KOG0371 Serine/threonine protein phosphatase 2A, catalytic subunit | 35 |
|  |  | [TR] KOG0374 Serine/threonine specific protein phosphatase PP1, catalytic subunit | 33.7893818544354 |
|  |  | [T] KOG0377 Protein serine/threonine phosphatase RDGC/PPEF, contains STphosphatase and EF-hand domains | 33.7295256836321 |
|  |  | [R] KOG0376 Serine-threonine phosphatase 2A, catalytic subunit | 23.9791979952384 |

#### Graphs (click to enlarge):

|  |  |
| --- | --- |
| BLAST of Query Sequence | Location of Ortholog Group Hits |
|  |  |

Contact: Andre Cavalcanti\_\_\_\_\_Last Modified September 14, 2010
